# Music, Math, and Working Memory: Magnetoencephalography Mapping of Brain Activation in Musicians

**DOI:** 10.3389/fnhum.2022.866256

**Published:** 2022-05-16

**Authors:** Ching-I Lu, Margaret Greenwald, Yung-Yang Lin, Susan M. Bowyer

**Affiliations:** ^1^Department of Communication Sciences and Disorders, Wayne State University, Detroit, MI, United States; ^2^Department of Neurology, Wayne State University, Detroit, MI, United States; ^3^Institute of Brain Science and Institute of Clinical Medicine, National Yang Ming Chiao Tung University, Taipei, Taiwan; ^4^Department of Critical Care Medicine, Taipei Veterans General Hospital, Taipei, Taiwan; ^5^Department of Neurology, Henry Ford Health System, Detroit, MI, United States; ^6^Department of Physics, Oakland University, Rochester, MI, United States

**Keywords:** working memory, musical transposing, calculation, music training, magnetoencephalography (MEG)

## Abstract

Musical transposing is highly demanding of working memory, as it involves mentally converting notes from one musical key (i.e., pitch scale) to another key for singing or instrumental performance. Because musical transposing involves mental adjustment of notes up or down by a specific amount, it may share cognitive elements with arithmetical operations of addition and subtraction. We compared brain activity during high and low working memory load conditions of musical transposing versus math calculations in classically trained musicians. Magnetoencephalography (MEG) was sensitive to differences of task and working memory load. Frontal-occipital connections were highly active during transposing, but not during math calculations. Right motor and premotor regions were highly active in the more difficult condition of the transposing task. Multiple frontal lobe regions were highly active across tasks, including the left medial frontal area during both transposing and calculation tasks but the right medial frontal area only during calculations. In the more difficult calculation condition, right temporal regions were highly active. In coherence analyses and neural synchrony analyses, several similarities were seen across calculation tasks; however, latency analyses were sensitive to differences in task complexity across the calculation tasks due to the high temporal resolution of MEG. MEG can be used to examine musical cognition and the neural consequences of music training. Further systematic study of brain activity during high versus low memory load conditions of music and other cognitive tasks is needed to illuminate the neural bases of enhanced working memory ability in musicians as compared to non-musicians.

## Introduction

Working memory is enhanced in musicians as compared to non-musicians (George and Coch, 2011), but neuroimaging studies of musicians have yielded little information about their brain activity during cognitive tasks with high versus low working memory load conditions. Similarly, there is insufficient detail about high versus low working memory demands of specific music tasks in the general debate about potential cognitive effects of music training (Nutley et al., 2014; Swaminathan et al., 2017).

Working memory is highly taxed in some music tasks; in others, the working memory demand is very low. If music training includes only low demands on working memory, then working memory is not likely to improve from the training, nor would it be expected to influence working memory function that supports a different cognitive behavior, such as math or reading. One music task that is demanding of working memory is musical transposing, which involves mentally converting notes from one musical key (i.e., pitch scale) to another key for singing or instrumental performance. Working memory demands are high during musical transposing because the target musical key must be stored temporarily while notes are manipulated. No studies of the cognitive effects of musical training have included transposing in the music training program, though this would be one means to assess whether music training involving high working memory load would improve working memory capacity.

Ongoing research into the cognitive and neural effects of training in music includes studies of experts and non-experts, and the longitudinal effects of exposure and training in children (Schön et al., 2002; Stewart et al., 2003). Music and math are separate cognitive domains, and separable from language (Ivanova et al., 2020), although there is some evidence they may share domain-general structural processing mechanisms with language (Van de Cavey and Hartsuiker, 2016; Nakai and Okanoya, 2018). There is evidence for a relationship between musical achievement and math achievement, usually associative (Holochwost et al., 2017) rather than causal (Hille and Schupp, 2015; Wallick, 1998). Recently, Bergee and Weingarten (2021) controlled for background variables that may influence achievement in music, math, and reading in children, and found that musical achievement did relate to math and reading achievement. However, following a meta-analytic review of studies of music training, Sala and Gobet (2020) concluded that music training has no impact on non-music cognitive skills and academic achievement. In studies of the effects of music training, greater specificity is needed in descriptions of the cognitive components that are highly active during the music training tasks, and whether the training tasks involve cognitive abilities from domains other than music.

Math, for example, is linked to the musical transposing task in that changing from one musical key to another is based on mental calculations that can involve addition or subtraction skills. A varying potential for unidirectional or bidirectional influence of learning in music and math tasks will depend on the cognitive components needed to accomplish each task, and the type and degree of overlap between these cognitive components across tasks. Transposing is one example of a music task in which the influences of music and math during training of transposing may be bidirectional.

In previous reports, we have shown that magnetoencephalography (MEG) is sensitive to differences in working memory load (Lu et al., 2019, 2021). By comparing brain activity in musical transposing of musical notation versus sight-reading (in which notes are played as written), we observed that the additional mental conversion required for transposing was linked to slowed activation of the ventral (fusiform gyrus) occipito-temporal stream of visual-spatial encoding, and to increased frontal lobe activation (Lu et al., 2021).

Further studies are needed to compare aspects of music, language and mathematical cognition, and brain activity that supports them in musicians and non-musicians. For example, studies using visual music and math tasks are needed to determine the relative roles of domain-general skills in working memory and spatial attention (Sligte et al., 2009) compared to domain-specific cognitive abilities in music or math.

Because musical transposing involves mental calculations for modifying musical keys, it may share cognitive elements with arithmetical operations of addition and subtraction. It is therefore of interest to compare the brain activity underlying simple arithmetic to that underlying the mental conversion of transposing in which notes are adjusted up or down by a specific amount. Further, imposing a requirement that participants hold a cue in working memory to perform the arithmetic and transposing tasks adds another level of similarity across the tasks and a way for working memory load to be manipulated up or down. By studying similarities and differences across the arithmetic and transposing tasks of higher and lower working memory load, we obtain clues as to how these cognitive tasks are related and how training in one (e.g., music training) could possibly affect the other (e.g., performance in math).

In the current paper, we report brain activity in trained musicians during math calculation tasks compared to musical transposing using MEG. We also examine the effects of working memory load across music and calculation tasks.

## Materials and Methods

### Participants

Twenty-one participants at Taipei Veterans General Hospital, Taipei, Taiwan who were able to read the Western musical notation system completed the study voluntarily with normal vision, hearing, motor and cognitive abilities. All of the data from one participant had to be discarded due to sleeping, and individual transposing and calculation task data from two participants had to be discarded due to noise; thus, data on all tasks were analyzed for 19 participants. All participants were female classically trained musicians (age range = 19–28; *x* = 23.71) with at least 10 years of musical instrumental training (range = 10–22 years; *x* = 16.71), including reading of standard musical notation. Potential participants were not included if their major instrument was a transposing instrument (i.e., an instrument that produces a higher or lower pitch than is shown in music notation written for it). For example, if a musicians’ major instrument is clarinet in B flat, they would always automatically lower two interval pitches while they read the score in G clef. Thus, long-term intensive experience in transposing in these individuals has the potential to alter patterns of brain activity during transposing as compared to other musicians. For the musicians who were included in this study, a transposing instrument was not the major instrument and, based on typical music training in Taiwan in which students are trained to read transposing scores from approximately the 3rd to the 12th grade, the average amount of experience in transposing for the participants included in this study was approximately 10 years.

All participants completed the Edinburgh Handedness Inventory (Oldfield, 1971), and laterality quotients indicated strong right-handed preference for all but one participant, who was ambidextrous. They also passed the Mini-Mental State Examination (MMSE; Folstein et al., 1975), which screened for cognitive impairment, and the digit span task (WAIS-III; Wechsler, 1997), which measured working memory storage capacity. All participants were within normal range. None had a history of neurological or psychiatric diseases, or developmental learning difficulties. Each participant provided a written informed consent prior the experiment. The study was approved by the Taipei Veterans General Hospital Human Research Review Board, Taipei.

### Experimental Design and Stimuli

A two-factor within-participant design was used: 2_within_ (stimuli: Musical notation versus Digits) × 2_within_ (task: Easier – one note or one single-digit versus Difficult-five notes or five-single digits), with location, amplitude, and latency of activation as dependent variables. Brain activation was observed during the four experimental tasks below. We previously discussed the results of the transposing tasks in comparison to musical sight-reading (Lu et al., 2021).

#### One Single-Digit Calculation (1D)

A written cue indicated a plus (+) or minus (−) single digit from 1 to 5 presented for 1,000 ms randomly (+ 1, + 2, + 3, + 4, + 5, −1, −2, −3, −4, −5). After a 1,000 ms blank screen, each stimulus (*n* = 60) was presented randomly for 1,500 ms plus 1,000 ms ISI. Each stimulus was a single digit from 0 to 9. During each presentation, the participant was to silently add to or subtract from the target stimulus based on the plus or minus cue and then silently name the correct answer, with no overt action (e.g., the cue + 4 followed by the target 8 would be silently named as 12). This task lasted 4 min 30 s.

#### Five Single-Digit Calculation (5D)

**FIGURE 1:**
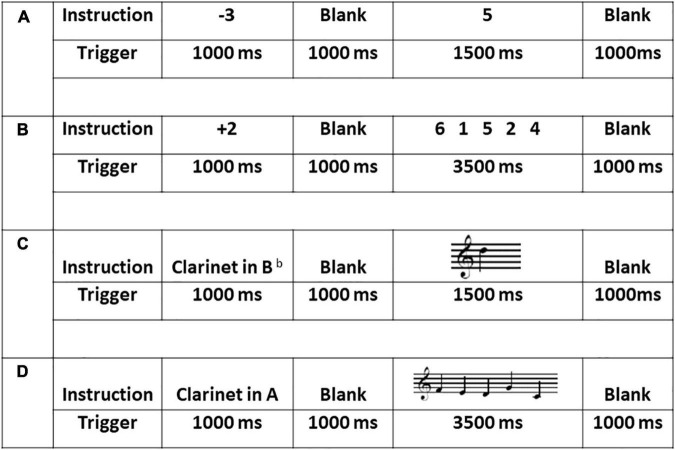
Sample stimuli, duration and procedure across the four experimental tasks: **(A)** the one single-digit calculation task (1D); **(B)** the five single-digit calculation task (5D); **(C)** the one-note transposing task (1T); and **(D)** the five-note transposing task (5T). All stimuli for 1T and 5T were presented on treble clef and all cues (transposing instruments) were presented using word form.

This task was identical to the one single-digit calculation task except that a sequence of five digits was presented for 3,500 ms (Figure 1). During each presentation, the participant was to silently add to or subtract from the target stimulus based on the plus or minus cue and then silently name the correct answer, with no overt action (e.g., the cue + 4 followed by the target 6 4 2 8 5 would be silently named, sequentially, as 10, 8, 6, 12, 9). This task lasted 6 min 30 s.

#### One-Note Transposing With Treble Clef (1T)

A written cue of one of five transposing instruments was presented for 1,000 ms pseudo randomly (Clarinet in A, in E^b^ or in B^b^, French horn in F, and Trumpet in B^b^). After 1,000 ms blank screen, each stimulus (*n* = 60) was presented randomly for 1,500 ms plus 1,000 ms ISI. During each presentation, the participant was to silently transpose from the written note to the target key and then silently name the new note, with no overt action. This task lasted 4 min 30 s.

#### Five-Note Transposing With Treble Clef (5T)

This task was identical to the 1T task except that a sequence of five notes was presented for 3,500 ms (Figure 1). During each presentation, the participant was to silently transpose each written note to the target key and then silently name the new notes sequentially, with no overt action. This task lasted 6 min 30 s.

### Procedure

Stimuli were presented electronically using E-Prime Professional 2.0 software (Psychology Software Tools, Pittsburgh, PA). To document participant accuracy in the tasks, behavioral practice data were collected using overt naming before each participant entered the MEG scanner; over 60 trials of each task, the average pre-test single accuracy was 99% for 1D, 95% for 5D, 87% for 1T, and 80% for 5T. Silent naming was required inside the scanner due to decreased signal noise (due to mouth movement) in silent naming compared to overt naming. Participant brain waves were monitored during tasks in the scanner to ensure participant alertness.

Inside the scanner, oral and written instructions were given immediately prior to each task, with 2-min breaks between tasks. The 1T task was given before the 5T task followed by the 1D and 5D tasks. Including screening, practice and experimental tasks, the procedure lasted approximately 90 min (Figure 1). Immediately following the MEG scan, each participant was asked a general open-ended question as to how they completed the transposing and math tasks.

### Data Acquisition and Preprocessing

A 306-channel MEG system (Vectorview, Elekta-Neuromag, Helsinki, Finland) was used; this helmet-shaped device covers the entire adult head except for the face. Participants were monitored continuously by intercom and camera. During data collection, participants were asked to avoid eye and body movements. MEG data were recorded with a high pass filter of 0.1 Hz, low pass filter of 100 Hz, and sampling rate of 508.63 Hz.

MEG signals measured the magnetic fields produced by currents fed into four head position indicator coils at known scalp locations, two high behind the earlobes and two wide apart high on the forehead. Coil locations were chosen in relation to three anatomical landmarks, including left preauricular point, right preauricular point, and nasion, which were determined with three-dimensional digitization. The individual sensors were magnetometers. Head shape was digitized for coregistration to the standard female brain template of T1-weighted MRI. The MRI scan was performed on a GE 1.5-T, 1-m-bore whole body magnet. MRI scan parameters were coronal T1 images, 124 slices, and 256 × 256 matrix including the entire skin surface of the head. A model of cortical brain surface was created from this standard MRI and performed in MEG-TOOLS (Moran et al., 2005). The MRI was segmented and brain surface was represented by a cortical model of approximately 4,000 dipoles each having x, y, and z orientation at each site. Sites were distributed to represent the same volume of cortical gray matter. This model was then morphed to fit the digitized head shape collected during MEG acquisition.

Using an independent component analysis (ICA), noise artifacts due to heart and body movement were eliminated in post-processing. Any other artifacts in the data were removed if needed using singular valued decomposition. Regarding movement artifact, runs would have been repeated if the coil on head positions exceeded 0.5 cm, although this did not occur during data acquisition. Data were filtered 3–85 Hz with notch at 60 Hz. The locations of events on trigger and response channels were used to select 1.5-s epochs of MEG data to examine average evoked responses during the four experimental tasks.

#### MR-FOCUSS

Event-related cortical activation was studied by averaging all 60 trials of the participant’s measured evoked MEG field responses during each task. Data were analyzed using MR-FOCUSS (current distribution technique; Moran et al., 2005) to localize and quantify cortical activation within the brain. The latency (in ms), location and average amplitude of response (nAm/time point) were extracted from MR-FOCUSS imaging results. MR-FOCUSS cortical mapping was applied to the interval 0–1,500 ms after stimulus onset in each experimental task. Selection of significant cortical activation was determined by visually inspecting imaged MEG solutions overlaid on the anatomical MRI and setting the display threshold to 30% (color coded blue) of the maximum cortical source amplitude (color coded red), and by selecting the high peaks of activity relative to the background brain noise.

##### Coherence Source Imaging

Synchronization of neuronal activity was quantified by calculating coherence between cortical sites from MEG imaged brain activation (Elisevich et al., 2011; Bowyer, 2016). A model of the cortical brain surface was created from an age- appropriate standard MRI of a female brain, as described above. To calculate coherence, the MEG data were first divided into 40 (1T or 1D tasks) or 52 (5T or 5D tasks) segments each containing 7.5-s segments of data, and cortical activity in each segment was imaged on the MRI using the MR-FOCUSS imaging technique. Using the time sequence of imaged activity, coherence between active cortical model sites was calculated for each data segment and then averaged for the completed study. In addition, for each cortical model site, connectivity was quantified by a histogram of the number of sites to which the site had the same level of coherence. Statistical analysis of cortical coherence levels (0 to 1) were used to quantify differences in network connectivity between groups. Changes in coherence and connectivity between brain regions implicated as having deviant electrophysiological activity in different tasks within the participants’ brains were quantified and included in further statistical analysis.

A region-of-interest (ROI) tool implemented in MEG Tools was used to identify 54 regions in the brain (27 in each hemisphere). MEG Tools uses a non-linear volumetric transformation of the participant’s brain to transform MEG coordinates to standard Talairach or MNI coordinates. This enables the ROI tool to access an atlas of Brodmann’s area identifiers and an atlas of cortical structures.

##### Neural Synchrony

*T*-test was used to assess task difference in average coherence values for each pair of brain regions (*N* = 1431) (Lajiness-O’Neill et al., 2014). A *p* value was produced for each region pair. Because of the large number of tests being performed simultaneously, using a significance level of alphas = 0.1 without adjusting for multiple testing would lead to a large number of false positive results; therefore, false discovery (FDR) was used to adjust for multiple testing. Bonferroni adjustments for multiple comparisons aim to control the family wise error rate. From each *t*-test, a *t*-score was computed according to the method of Efron to summarize the difference in coherence values between tasks.

## Results

### Latency and Amplitude

**FIGURE 2:**
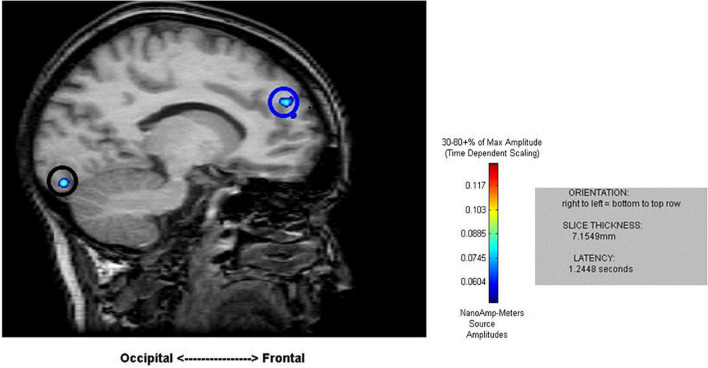
Sample results of the MEG recordings (MR-FOCUSS analyses) for an individual participant showing simultaneous frontal and occipital activation at 1,244 ms latency in five-note transposing (5T).

**FIGURE 3:**
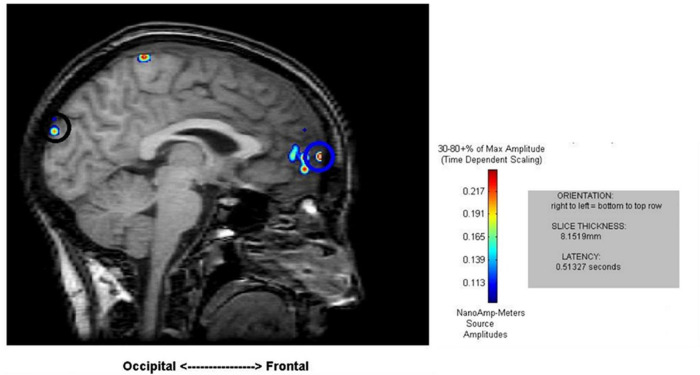
Sample results of the MEG recordings (MR-FOCUSS analyses) showing simultaneous frontal and occipital activation at 513 ms latency in five single-digit calculation (5D) for the same participant shown in Figure 2.

Cortical mapping using MR-FOCUSS analysis displayed multiple areas of neuronal activity, including visual cortex, fusiform gyrus, superior temporal gyrus (STG), angular gyrus (AG), supramarginal gyrus (SMG; Wernicke’s area included activation of the STG, AG, and SMG), the superior parietal gyrus, and frontal lobe regions. Simultaneous activation of both visual and frontal gyri was also measured. Selected images from an individual participant are shown as examples in Figures 2, 3. Note the latencies for this individual fall within the midrange of the average across all subjects (showing simultaneous frontal and occipital activation occurring earlier for the 5D task than for the 5T task). The temporal resolution of peak activation in these areas during the four tasks is summarized in Table 1.

**TABLE 1:** Temporal resolution of magnetoencephalography (MEG) signals arising from the peak brain activation during each task

	Visual cortex	Fusiform gyrus	Superior parietal gyrus	Wernicke’s area^a^	Frontal and visual cortex^b^
One single-digit calculation (1D)	84 ± 14 ms	244 ± 131 ms	241 ± 107 ms	274 ± 99 ms	548 ± 103 ms
One-note transposing (1T)	83 ± 20 ms	237 ± 144 ms	216 ± 72 ms	199 ± 68 ms	968 ± 176 ms
Five single-digit calculation (5D)	91 ± 14 ms	236 ± 142 ms	257 ± 91 ms	282 ± 111 ms	666 ± 113 ms
Five-note transposing (5T)	84 ± 16 ms	241 ± 129 ms	270 ± 122 ms	271 ± 60 ms	1019 ± 127 ms

*^a^Bilateral superior temporal gyrus (STG), angular gyrus (AG), and supramarginal gyrus (SMG).*

*^b^Frontal and visual regions activated simultaneously.*

*ms, milliseconds.*

To better understand the three main effects [ROI (region of interest), task, memory load and their interaction effects], a three-factor repeated-measure analysis was conducted 5_within_ (ROI: visual versus fusiform versus Wernicke’s area versus superior parietal versus visual + frontal areas) × 2_within_ (task: calculation versus transposing) × 2_within_ (memory loading: one versus five) with peak latency of activity (visual, fusiform, superior parietal, Wernicke’s area, and visual + frontal) as dependent variables. Using general liner model – repeated measure revealed significant main effects of ROI [*F*(4,68) = 360.24, *p* < *0.001*], task [*F*(1,17) = 51.00, *p* < *0.001*], and loading [*F*(1,17) = 6.45, *p* = *0.021*]. There is an interaction effect between ROI and task [*F*(4,68) = 67.84, *p* < *0.001*]. Main effects of ROI showed no significant difference in early latency of activity across the four tasks (including visual, fusiform, and superior parietal). However, the four tasks showed significantly different latency at Wernicke’s area [*F*(372) = 3.64, *p* = *0.017*] and at the stage of simultaneous visual and frontal activation [*F*(3,72) = 56.35, *p* < *0.001*]. *Post hoc* comparisons of 1D versus 1T showed a significant difference at Wernicke’s area [*t* (18) = 3.47, *p* = *0.003*] such that 1T (199 ± 68 ms) was faster than 1D (274 ± 99 ms); for the 1T versus 5T comparison [*t* (17) = −3.38, *p* = *0.004*], 1T (202 ± 69 ms) was faster than 5T (272 ± 61 ms). At the stage of simultaneous visual and frontal activation), a significant difference was indicated for the 1D versus 1T comparison [*t* (18) = −10.12, *p* < *0.001*] such that 1D (548 ± 103 ms) was faster that 1T (968 ± 176 ms), for the 1D versus 5D comparison [*t* (18) = −3.05, *p* = *0.007*] such that 1D (548 ± 103 ms) was faster than 5D (666 ± 113 ms), and for the 5D versus 5T comparison [*t* (17) = −8.62, *p* < *0.001*] such that 5D (668 ± 117 ms) was faster than 5T (1028 ± 125 ms).

### Coherence Source Imaging

We analyzed the entire dataset for each task to find the top five highest coherence regions active for all of the participants combined (Table 2). During 1D, 5D, and 1T tasks, the highest coherent region was left parahippocampus; in 5T, the highest coherent region was right precentral motor cortex. Interestingly, the transposing tasks and calculation tasks all engaged the left medial frontal area. Also, the calculation tasks had the same top three highest coherent regions: Left parahippocampus, right medial frontal, and then left medial frontal.

**TABLE 2:** Spatial resolution of magnetoencephalography (MEG) signals arising from the top five highest coherent regions during each task.

	The highest region	2nd	3rd	4th	5th
One single-digit calculation (1D)	Left parahippocampus	Right medial frontal	Left medial frontal	Left inferior frontal	Right superior frontal
One-note transposing (1T)	Left parahippocampus	Left superior parietal	Right superior frontal	Right medial orbitofrontal	Left medial frontal
Five single-digit calculation (5D)	Left parahippocampus	Right medial frontal	Left medial frontal	Right middle temporal	Right fusiform
Five-note transposing (5T)	Right precentral (BA4)	Right superior occipital	Right inferior frontal	Right precentral (BA6)	Left medial frontal

### Neural Synchrony Analysis

To identify neuronal networks most strongly activated during each task, 1,431 pathway connections were evaluated for their coherence value in each task, and then a bootstrap method was used to identify differences (*p* < 0.05) between tasks. This provides information on which networks (i.e., two locations) are significantly involved for each task compared to the other task.

In comparing the 5D networks versus the 5T networks, significant differences were found in 13 out of 1,431 pathways. In particular, inter-hemispheric network activity differences were identified between limbic system and other regions. The *p* values of less than 0.05 identified using this procedure are listed in Table 3.

**TABLE 3:** Differences in network activation: Five single-digit calculation (5D) versus five-note transposing (5T).

Path	Mean.5D	Mean.5T	*t*	*p*.value
L.cingulate_gyrus.L.inferior_frontal_gyrus	0.027	0.021	2.109	0.042
R.cingulate_gyrus.R.gyrus_rectus	0.03	0.02	2.057	0.047
L.inferior_occipital_gyrus.R.parahippocampal_gyrus	0.053	0.073	–2.586	0.014
L.inferior_occipital_gyrus.R.insular_cortex	0.028	0.04	–2.265	0.03
R.parahippocampal_gyrus.R.supramarginal_gyrus	0.037	0.049	–2.192	0.035
L.inferior_occipital_gyrus.R.angular_gyrus	0.163	0.197	–2.18	0.036
L.inferior_occipital_gyrus.R.superior_temporal_gyrus	0.135	0.167	–2.131	0.04
R.insular_cortex.R.lingual_gyrus	0.014	0.02	–2.128	0.04
L.lateral_orbitofrontal_gyrus.R.insular_cortex	0.022	0.03	–2.098	0.043
L.inferior_occipital_gyrus.R.supramarginal_gyrus	0.155	0.187	–2.084	0.044
R.angular_gyrus.R.parahippocampal_gyrus	0.038	0.05	–2.084	0.044
L.lingual_gyrus.R.insular_cortex	0.015	0.021	–2.039	0.049
L.middle_temporal_gyrus.R.insular_cortex	0.024	0.031	–2.027	0.05

*The limbic system including insula, putamen, parahippocampal gyrus, caudate, hippocampus.*

**FIGURE 4:**
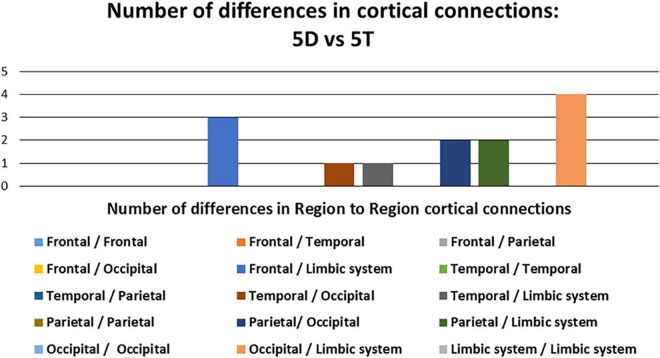
Number of differences in neural connections: Comparison between five single-digit calculation (5D) versus five-note transposing (5T).

Of the 13 pathways that were found to have significant differences between the 5D networks versus the 5T networks, the most likely (i.e., most common) was the occipital-limbic system pathway. This is shown in Figure 4, as compared to the less likely pathways.

In comparing the 1D networks versus the 1T networks, significant differences were found in 3 out of 1,431 pathways. In particular, inter-hemispheric network activity differences were identified between occipital and frontal regions.

Of the 3 pathways that were found to have significant differences between the 1D networks versus the 1T networks, the most likely (i.e., most common) was the frontal-occipital pathway, especially in right inferior frontal gyrus. The negative *t* values showed that the 1T networks are more active than 1D networks between the occipital and frontal regions (Table 4).

**TABLE 4:** Differences in network activation: One single-digit calculation (1D) versus one note transposing (1T).

Path	Mean.1D	Mean.1T	*T*	*p*.value
L.inferior_occipital_gyrus. R.inferior_frontal_gyrus	0.185	0.22	−2.244	0.031
L.superior_occipital_gyrus. R.inferior_frontal_gyrus	0.133	0.163	−2.074	0.045
L.middle_occipital_gyrus. R.inferior_frontal_gyrus	0.178	0.21	−2.062	0.046

The 1D networks versus the 5D networks were compared. Significant differences were found in none of 1,431 pathways.

In neural synchrony analysis, 2 of the 1,431 pathways differed significantly between the 5T versus 1T. Strong network differences between these transposing tasks were observed in connections from left superior occipital gyrus to left frontal regions (Table 5). As indicated by the positive t values, the 5T task involved these pathways significantly more than 1T.

**TABLE 5:** Differences in network activation: Five-note transposing (5T) versus one-note transposing (1T).

Path	Mean.5T	Mean.1T	*T*	*p*.value
L.inferior_frontal_gyrus. L.superior_occipital_gyrus	0.147	0.121	2.877	0.007
L.precentral_gyrus. L.superior_occipital_gyrus	0.17	0.147	2.025	0.05

**FIGURE 5:**
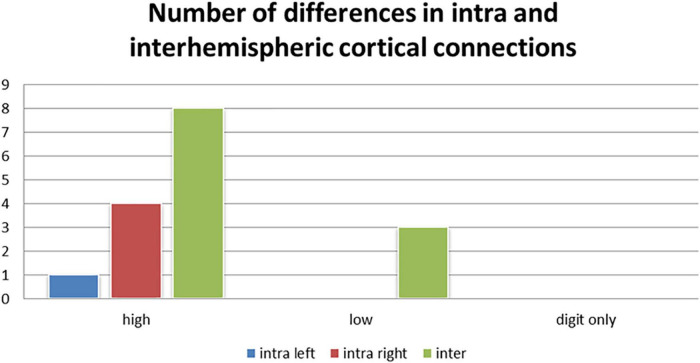
Number of differences in neural connections: Intra-right hemisphere, intra-left hemisphere and inter-hemispheric cortical differences in tasks of high working memory load (5D versus 5T), low working memory load (1D versus 1T), and digits only (5D versus 1D).

We also observed the following intra- and inter-hemispheric differences in coherence across tasks of calculation versus transposing of high and low working memory load (5D versus 5T, 1D versus 1T; Figure 5). Of the pathways found to have significant differences between the two tasks of high working memory load (5D versus 5T), the most likely pathways were inter-hemispheric.

After the MEG session, each participant responded to an informal query about their strategy in the experimental tasks. The participants reported that they responded one-by-one to the five digits or notes presented in each 5D and 5T stimulus; further information about individual strategy was not obtained.

## Discussion

MEG is sensitive to differences in brain activation in musical transposing versus digit calculation, as shown in this study, and can be used to examine the neural correlates of musical and mathematical cognition and the consequences of music training. The patterns of brain activation observed here are influenced by working memory load and task type.

Transposing differed from calculation in frontal-occipital activation. The simultaneous frontal and occipital activation occurred significantly more slowly during transposing compared to calculation (1T slower than 1D; 5T slower than 5D). Neural synchrony analyses revealed more frontal-occipital neural connections active in 5T than 5D, and in 1T than in 1D. Frontal-occipital interactions support visual perception (Ruff et al., 2006) and visual working memory (Barton and Brewer, 2013). In the current study, the notes to be transposed are perceived among multiple elements that indicate clef, staff, and key signature, whereas in calculation the viewed digits form a simpler display. In transposing, high demands on visual working memory and visual perception may have required greater support from visual cortex and its interactions with frontal lobe systems.

The 5T task involved left occipital-frontal network pathways significantly more than the 1T, and the top four most active regions in 5T were in frontal or occipital areas. Greater visual complexity of the visual pre-cue cannot account for increased frontal-occipital activation in the transposing tasks versus calculation, because previously we identified high activity in frontal-occipital areas in both high and low memory load conditions of musical sight-reading wherein there is no pre-cue (Lu et al., 2021). Musical sight-reading stimuli were similar in complexity to transposing stimuli; as in the 5T task, the high- and low-load sight-reading tasks resulted in high activity in frontal lobe areas and right superior occipital gyrus. In contrast, occipital cortex and frontal-occipital connections were not highly activated in the calculation task.

Interestingly, among the top three most highly active areas in 5T were right primary motor cortex (BA4) and right premotor cortex and supplementary motor area (BA6), possibly reflecting motor planning or programming of hand movement (consciously or unconsciously) when musical notation was viewed. Right precentral cortex was also found to be highly active in both high- and low-load sight-reading (Lu et al., 2021). High activity in the motor cortex was not evident during calculation, suggesting the digit stimuli did not result in initiation of motor programming.

In both the coherence analyses (in top three most highly active regions) and the neural synchrony analyses, the calculation tasks were found to have similar patterns of brain activation. However, in latency analysis there was a significantly slower peak of simultaneous frontal and occipital activation in 5D compared to 1D. This likely reflects increased working memory demands in 5D compared to 1D, detected by the high temporal resolution of MEG.

Parahippocampal gyrus, part of medial temporal lobe and the limbic system, was the most highly active brain area for the calculation tasks and 1T. This area is involved in visuospatial processing and other cognitive functions such as memory (Aminoff et al., 2013). In neural synchrony comparisons, the 5D task involved the occipital-limbic pathway significantly more than did 5T. Limbic activity was not as prominent in the 5T task, which had high activations in other regions such as right motor and premotor cortex.

Multiple distributed processes and brain regions have been described as contributing to working memory; for example, mental representations encoding visual feature information in temporal-occipital cortex (ventral visual pathway), spatial information in frontal-parietal cortex (dorsal visual pathway), and information such as behavioral significance in frontal cortex (Eriksson et al., 2015; Ren et al., 2019). The right medial temporal lobe provides important support for memory (Jeneson and Squire, 2012), and the fusiform gyrus is involved in higher visual perception and memory (Weiner et al., 2017). In the current study, when the calculation task involved more numbers to be added or subtracted the participants relied more on right medial temporal lobe and right fusiform gyrus, both highly active in 5D but not in 1D. Previously, we (Lu et al., 2021) attributed slowed fusiform activation in transposing compared to musical sight-reading to the additional mental conversion required for transposing; this may require more ventral stream than dorsal stream processing. Interestingly, in the current study with all four tasks more highly demanding of working memory than the musical sight-reading task, there is no significant difference in time course of fusiform activation.

Superior parietal cortex is important for spatial encoding (Stewart, 2005), and was highly active in the left hemisphere during 1T in the current study. As part of a dorsal “where” stream of spatial encoding, this region is involved in encoding stimulus location, whereas a ventral “what” stream that includes the fusiform appears to encode features for stimulus identification (Mishkin and Ungerleider, 1982; Goodale and Milner, 1992). Reading music may involve interaction between the dorsal and ventral streams (Mongelli et al., 2017). Previously, we (Lu et al., 2019) identified bilateral activation of superior parietal cortex during silent reading of English letters and musical notes. In the current study this region likely played a role across tasks but was not prominently activated in 5T, 5D and 1D.

The left medial frontal area was highly active during all four experimental tasks; interestingly, right medial frontal area was highly active during calculation tasks but not during transposing tasks. Functions of frontal lobe regions may overlap in supporting working memory (Duncan and Owen, 2000); within this distribution of function the right medial frontal area may have some relative specialization for supporting calculation. Differences in working memory demands across tasks have influenced efforts to define frontal lobe specialization in mathematical tasks; for example, Hayashi and colleagues (Hayashi et al., 2000) observed activation of right frontal areas during subtraction but not multiplication, which they attributed to greater working memory demands in their subtraction task. In the current study, the task design was the same for the transposing and calculation tasks; nevertheless, differences in visual display and the nature of mental conversion needed to solve each item may have placed more demands on working memory in transposing compared to math calculations.

Along with left and right medial frontal areas, several other frontal lobe regions were highly active in the current experimental tasks. The right inferior frontal lobe has been described as important for supporting mathematical subtraction (Kong et al., 2005). These results, along with high activity of the right inferior frontal area in the 5T task, occipital connections to right inferior frontal cortex in the 1T task (shown in neural synchrony analysis), and high activity in left inferior frontal area in the 1D task suggest that these inferior frontal areas should be examined further in comparisons of neural activity in music versus math. The specific roles of the right superior frontal gyrus (highly active in both 1D and 1T), and the right medial orbitofrontal area (highly active in 1T) in working memory also need further study as they relate to musical and mathematical cognition. Given that activation patterns of frontal lobe and frontal-posterior networks can be influenced by specific modifications to task design within domain (e.g., differential effects of a pre-cue versus a post-cue; Ruff et al., 2007), more research is needed to define how task adjustments within and across music and math tasks may affect similarities and differences in corresponding neural activity.

Neural responses elicited during the calculation tasks in the current study are affected by the high working memory demands of the tasks. Using an arithmetic equation verification task in which working memory demands were limited, Rosenberg-Lee and colleagues (Rosenberg-Lee et al., 2011) assessed neural responses in tasks of addition, subtraction, multiplication and division. Among the brain regions they identified as involved in these tasks, the posterior parietal cortex (PPC) was shown to be critically involved in representing cognitive processes of retrieval, calculation and inversion differentially involved in these tasks. Others have identified the PPC as important in supporting mathematical functions (Dehaene and Cohen, 1997). In the current study, MEG was sensitive to differences in high and low working memory load across tasks, as described above. However, all of the tasks were demanding of working memory and did result in high activity in brain regions supporting working memory and, overall, relatively lower activation of brain regions supporting other cognitive elements of the tasks.

In the present investigation, though participants were required to silently name the solutions to each transposing or math problem (e.g., “B” or “7” for the 1T and 1D tasks, and sequentially during the 5T and 5D tasks), left hemisphere activation was not dominant during these tasks. This is in contrast to the results of laterality index analyses in our previous study (Lu et al., 2019) in which silent naming of English letters and silent reading of notes in musical sight-reading both resulted in left hemisphere-dominant activation. We (Lu et al., 2019) hypothesized that those results reflected left hemisphere phonological activation to support silent naming based on prior findings that MEG neural activity supporting silent naming is lateralized to the left hemisphere in right-handed individuals (Bowyer et al., 2004). In the current study, results from the three analysis methods used do not show that brain activation is more lateralized to the left hemisphere for the transposing or calculation tasks. This is another example of how high task demands on working memory here may have eclipsed other cognitive elements of the tasks that can be observed using MEG when the working memory task demands are lower.

Expert musicians, and students being trained in musical transposing, may employ one or more strategies to accomplish transposing during ongoing performance. We previously described potential differences in musical transposing strategies that may include reliance on auditory imagery, visual-spatial imagery, or both (Lu et al., 2021). The activation of motor cortex during the 5T task in the current study may reflect a motor strategy in transposing. It remains unclear whether this motor activation reflects motor intention or occurred unconsciously in these highly trained musicians presented with musical notation. Further research is warranted into individual transposing strategies used by musicians, and by students during their development of transposing skills. The possibility of strategic motor involvement in transposing could be examined for potential differences in motor activation in response to notes presented in the treble clef (associated with right hand movement in piano) versus bass clef (associated with left hand movement in piano).

Current research into whether music training can affect math achievement includes hypotheses about the effects of music training on domain-general processes such as working memory (Eriksson et al., 2015). These effects have been examined in relation to subtypes of working memory; for example, Roden and colleagues (Roden et al., 2012) found that music training resulted in no improvement in visual memory, but that verbal memory improved. Simmons and colleagues (Simmons et al., 2012) described that subcomponents of working memory have different relationships with different mathematical skills. Continued research into cognitive elements involved in easier and harder iterations of music and math tasks (and cognitive elements shared across tasks) will influence efforts to specify the neural correlates of these cognitive subcomponents. MEG tasks with lower working memory load may allow observation of neural activity supporting specific cognitive elements that are not seen when working memory load is high; however, an advantage of using tasks with high memory load is that they may be closer to conditions in the real world. In real world performance of music or math, working memory demands will vary with the task but will be generally high. For example, during real world musical performance, the broader influences of context and attentional demands along with the musician’s work to interpret meaning and emotion all contribute to task complexity. With increasing expertise in music or math, information processing load will be reduced in some aspects, such as greater ability to use context to anticipate continuations in music (Waters et al., 1997). Along with ongoing advances in technology and data analysis methods, systematic adjustments of cognitive task demands on working memory and other cognitive subcomponents will be needed to capture how the brain supports music and math functions in experts and non-experts, and the cognitive effects of training.

## Conclusion

MEG was sensitive to differences in working memory load during musical transposing and calculation tasks in a group of classically trained musicians. Frontal-occipital connections were highly active during the transposing tasks, but not during the math calculation tasks. Right temporal regions were highly active in the more difficult condition of the calculation task. Multiple frontal lobe regions were highly active across tasks; notably, the left medial frontal area was highly active in all four tasks, but the right medial frontal area was highly active only during calculations. Right motor and premotor regions were highly active in the more difficult condition of the transposing task but not during calculations. Coherence analyses and neural synchrony analyses yielded several similarities in brain activation across the calculation tasks, but latency analyses were sensitive to differences in task complexity across the two tasks due to the high temporal resolution of MEG. As was done in the current study, future studies should compare brain activity in cognitive tasks involving higher versus lower working memory load. Systematic manipulations to task demands on working memory and other cognitive elements of music and math tasks will be necessary to specify the brain regions supporting elements of these tasks in experts and non-experts. MEG is sensitive to these effects and can be used to examine the neural correlates of musical and mathematical cognition and the consequences of music training.

## Data Availability Statement

The datasets presented in this article are not readily available as the format is unique. Requests to access the datasets should be directed to C-IL, chingilu@gmail.com.

## Ethics Statement

The studies involving human participants were reviewed and approved by Taipei Veterans General Hospital Human Research Review Board, Taipei. The patients/participants provided their written informed consent to participate in this study.

## Author Contributions

C-IL, MG, and SB contributed to conception and design of the study. C-IL and Y-YL contributed to study procedures, and collection and organization of data. C-IL and SB processed the data. C-IL performed the statistical analyses. C-IL and MG wrote sections of the manuscript. MG wrote the first draft of the manuscript. All authors contributed to manuscript revision, read, and approved the submitted version.

## Conflict of Interest

The authors declare that the research was conducted in the absence of any commercial or financial relationships that could be construed as a potential conflict of interest. The reviewer EWP declared a past co-authorship with one of the authors SB to the handling editor.

## Publisher’s Note

All claims expressed in this article are solely those of the authors and do not necessarily represent those of their affiliated organizations, or those of the publisher, the editors and the reviewers. Any product that may be evaluated in this article, or claim that may be made by its manufacturer, is not guaranteed or endorsed by the publisher.

## References

[B1] Aminoff E. M. Kveraga K. Bar M. (2013). The role of the parahippocampal cortex in cognition. *Trends Cogn. Sci.* 17 379–390. 10.1016/j.tics.2013.06.009 23850264 PMC3786097

[B2] Barton B. Brewer A. A. (2013). Visual working memory in human cortex. *Psychology(Irvine)* 4 655–662. 10.4236/psych.2013.48093 26881188 PMC4752675

[B3] Bergee M. Weingarten K. M. (2021). Multilevel models of the relationship between music achievement and reading and math achievement. *J. Res. Music Educ.* 68 398–418. 10.1177/0022429420941432

[B4] Bowyer S. M. (2016). Coherence a measure of the brain networks: past and present. *Neuropsychiatr. Electrophysiol.* 2:1. 10.1186/s40810-015-0015-7

[B5] Bowyer S. M. Moran J. E. Mason K. M. Constantinou J. E. Smith B. J. Barkley G. L. (2004). MEG localization of language-specific cortex utilizing MR-FOCUSS. *Neurology* 62 2247–2255. 10.1212/01.wnl.0000130385.21160.7a 15210890

[B6] Dehaene S. Cohen L. (1997). Cerebral pathways for calculation: double dissociation between rote verbal and quantitative knowledge of arithmetic. *Cortex* 33 219–250. 10.1016/s0010-9452(08)70002-9 9220256

[B7] Duncan J. Owen A. M. (2000). Common regions of the human frontal lobe recruited by diverse cognitive demands. *Trends Neurosci.* 23 475–483. 10.1016/S0166-2236(00)01633-7 11006464

[B8] Elisevich K. Shukla N. Moran J. E. Smith B. Schultz L. Mason K. (2011). An assessment of MEG coherence imaging in the study of temporal lobe epilepsy. *Epilepsia* 52 1110–1119. 10.1111/j.1528-1167.2011.02990.x 21366556 PMC3116050

[B9] Eriksson J. Vogel E. K. Lansner A. Bergstrom F. Nyberg L. (2015). Neurocognitive architecture of working memory. *Neuron* 88 33–46. 10.1016/j.neuron.2015.09.020 26447571 PMC4605545

[B10] Folstein M. F. Folstein S. E. McHugh P. R. (1975). Mini-Mental state: a practical method for grading the cognitive state of patients for the clinician. *J. Psychiatr. Res.* 12 189–198. 10.1016/0022-3956(75)90026-6 1202204

[B11] George E. M. Coch D. (2011). Music training and working memory: an ERP study. *Neuropsychologia* 49 1083–1094. 10.1016/j.neuropsychologia.2011.02.001 21315092

[B12] Goodale M. A. Milner A. D. (1992). Separate visual pathways for perception and action. *Trends Neurosci.* 15 20–25. 10.1016/0166-2236(92)90344-8 1374953

[B13] Hayashi N. Ishii K. Kitagaki H. Kazui H. (2000). Regional differences in cerebral blood flow during recitation of the multiplication table and actual calculation: a positron emission tomography study. *J. Neurol. Sci.* 176 102–108. 10.1016/S0022-510X(00)00323-3 10930591

[B14] Hille A. Schupp J. (2015). How learning a musical instrument affects the development of skills. *Econ. Educ. Rev.* 44 56–82. 10.1016/j.econedurev.2014.10.007

[B15] Holochwost S. J. Propper C. B. Wolf D. P. Willoughby M. T. Fisher K. R. Kolacz J. (2017). Music education, academic achievement, and executive functions. *Psychol. Aesthet. Creat. Arts* 11 147–166. 10.1037/aca0000112

[B16] Ivanova A. A. Srikant S. Sueoka Y. Kean H. H. Dhamala R. O’Reilly U. (2020). Comprehension of computer code relies primarily on domain-general executive brain regions. *Elife* 9:e58906. 10.7554/eLife.58906 33319744 PMC7738192

[B17] Jeneson A. Squire L. (2012). Working memory, long-term memory, and medial temporal lobe function. *Learn. Mem.* 19 15–25. 10.1101/lm.024018.111 22180053 PMC3246590

[B18] Kong J. Wang C. Kwong K. Vangel M. Chua E. Gollub R. (2005). The neural substrate of arithmetic operations and procedure complexity. *Cogn. Brain Res.* 22 397–405. 10.1016/j.cogbrainres.2004.09.011 15722210

[B19] Lajiness-O’Neill R. Richard A. E. Moran J. E. Olszewski A. Pawluk L. Jacobson D. (2014). Neural synchrony examined with magnetoencephalography (MEG) during eye gaze processing in autism spectrum disorders: preliminary findings. *J. Neurodev. Disord.* 6:15. 10.1186/1866-1955-6-15 24976870 PMC4072845

[B20] Lu C. Greenwald M. L. Lin Y. Bowyer S. M. (2019). Reading musical notation versus english letters: mapping brain activation with MEG. *Psychol. Music* 47 255–269. 10.1177/0305735617744886

[B21] Lu C. Greenwald M. L. Lin Y. Bowyer S. M. (2021). Musical transposing versus sight-reading: mapping brain activation with Magnetoencephalography (MEG). *Psychol. Music* 49 581–599. 10.1177/0305735619883692

[B22] Mishkin M. Ungerleider L. G. (1982). Contribution of striate inputs to the visuospatial functions of parieto-preoccipital cortex in monkeys. *Behav. Brain Res.* 6 57–77. 10.1016/0166-4328(82)90081-X 7126325

[B23] Mongelli V. Dehaene S. Vinckier F. Peretz I. Bartolomeo P. Cohen L. (2017). Music and words in the visual cortex: the impact of musical expertise. *Cortex* 86 260–274. 10.1016/j.cortex.2016.05.016 27317491

[B24] Moran J. E. Bowyer S. M. Tepley N. (2005). Multi-resolution FOCUSS: a source imaging technique applied to MEG data. *Brain Topogr.* 18 1–17. 10.1007/s10548-005-7896-x 16193262

[B25] Nakai T. Okanoya K. (2018). Neural evidence of cross-domain structural interaction between language and arithmetic. *Sci. Rep.* 8:12873. 10.1038/s41598-018-31279-8 30150643 PMC6110712

[B26] Nutley S. B. Darki F. Klingberg T. (2014). Music practice is associated with development of working memory during childhood and adolescence. *Front. Hum. Neurosci.* 7:926. 10.3389/fnhum.2013.00926 24431997 PMC3882720

[B27] Oldfield R. C. (1971). The assessment and analysis of handedness: the edinburgh inventory. *Neuropsychologia* 9 97–113. 10.1016/0028-3932(71)90067-4 5146491

[B28] Ren Z. Zhang Y. He H. Feng Q. Bi T. Qui J. (2019). The different brain mechanisms of object and spatial working memory: voxel-based morphometry and resting-state functional connectivity. *Front. Hum. Neurosci.* 13:248. 10.3389/fnhum.2019.00248 31379543 PMC6659551

[B29] Roden I. Kreutz G. Bongard S. (2012). Effects of a school-based instrumental music program on verbal and visual memory in primary school children: a longitudinal study. *Front. Psychol.* 3:572. 10.3389/fpsyg.2012.00572 23267341 PMC3528082

[B30] Rosenberg-Lee M. Chang T. T. Young C. B. Wu S. Menon V. (2011). Functional dissociations between four basic arithmetic operations in the human posterior parietal cortex: a cytoarchitectonic mapping study. *Neuropsychologia* 49 2592–2608. 10.1016/j.neuropsychologia.2011.04.035 21616086 PMC3165023

[B31] Ruff C. C. Blankenburg F. Bjoertomt O. Bestmann S. Freeman E. Haynes J. D. (2006). Concurrent TMS-fMRI and psychophysics reveal frontal influences on human retinotopic visual cortex. *Curr. Biol.* 8 1479–1488. 10.1016/j.cub.2006.06.057 16890523

[B32] Ruff C. C. Kristjansson A. Driver J. (2007). Readout from iconic memory and selective spatial attention involve similar neural processes. *Psychol. Sci.* 18 901–909. 10.1111/j.1467-9280.2007.01998.x 17894608 PMC2440528

[B33] Sala G. Gobet F. (2020). Cognitive and academic benefits of music training with children: a multilevel meta-analysis. *Mem. Cognit.* 48 1429–1441. 10.3758/s13421-020-01060-2 32728850 PMC7683441

[B34] Schön D. Anton J. L. Roth M. Besson M. (2002). An fMRI study of music sight-reading. *Neuro Rep.* 13 2285–2289. 10.1097/01.wnr.0000044224.79663.f5 12488812

[B35] Simmons F. Willis C. Adams A. (2012). Different components of working memory have different relationships with different mathematical skills. *J. Exp. Child Psychol.* 111 139–155. 10.1016/j.jecp.2011.08.011 22018889

[B36] Sligte L. G. Scholte H. S. Lamme V. A. F. (2009). V4 activity predicts the strength of visual short-term memory representations. *J. Neurosci.* 29 7432–7438. 10.1523/JNEUROSCI.0784-09.2009 19515911 PMC6665414

[B37] Stewart L. (2005). A neurocognitive approach to music reading. *Ann. N. Y. Acad. Sci.* 1060 377–386. 16597789 10.1196/annals.1360.032

[B38] Stewart L. Henson R. Kampe K. Walsh V. Turner R. Frith U. (2003). Brain changes after learning to read and play music. *Neuroimage* 20 71–83. 10.1016/S1053-8119(03)00248-9 14527571

[B39] Swaminathan S. Schellenberg E. Khalil S. (2017). Revisiting the association between music lessons and intelligence: training effects or music aptitude? *Intelligence* 62 119–124. 10.1016/j.intell.2017.03.005

[B40] Van de Cavey J. Hartsuiker R. (2016). Is there a domain-general cognitive sequencing system? Evidence from structural priming across music, math, action descriptions, and language. *Cognition* 146 172–184. 10.1016/j.cognition.2015.09.013 26414249

[B41] Wallick M. (1998). A comparison of ohio proficiency test results between fourth-grade pullout students and those of matched ability. *J. Res. Music Educ.* 46 239–247. 10.2307/3345626

[B42] Waters A. J. Underwood G. Findlay J. M. (1997). Studying expertise in music reading: use of a pattern-matching paradigm. *Percept. Psychophys.* 59 477–488. 10.3758/BF03211857 9158323

[B43] Wechsler D. (1997). *Wechsler Adult Intelligence Scale*, 3rd Edn. San Antonio, TX: The Psychological Corp.

[B44] Weiner K. S. Barnett M. A. Lorenz S. Caspers J. Stigliani A. Amunts K. (2017). The cytoarchitecture of domain-specific regions in human high-level visual cortex. *Cereb. Cortex* 27 146–161. 10.1093/cercor/bhw361 27909003 PMC5939223

